# The effect of dilution on eco‐evolutionary dynamics of experimental microbial communities

**DOI:** 10.1002/ece3.8065

**Published:** 2021-09-07

**Authors:** Thomas Scheuerl, Veijo Kaitala

**Affiliations:** ^1^ Department of Plant Sciences University of Cambridge Cambridge UK; ^2^ Research Department for Limnology University of Innsbruck Mondsee Austria; ^3^ Organismal and Evolutionary Biology Research Programme University of Helsinki Helsinki Finland

**Keywords:** ecological dynamics, evolutionary interaction, predator‐prey coexistence

## Abstract

Changing environmental conditions can infer structural modifications of predator‐prey communities. New conditions often increase mortality which reduces population sizes. Following this, predation pressure may decrease until populations are dense again. Dilution may thus have substantial impact not only on ecological but also on evolutionary dynamics because it amends population densities. Experimental studies, in which microbial populations are maintained by a repeated dilution into fresh conditions after a certain period, are extensively used approaches allowing us to obtain mechanistic insights into fundamental processes. By design, dilution, which depends on transfer volume (modifying mortality) and transfer interval (determining the time of interaction), is an inherent feature of these experiments, but often receives little attention. We further explore previously published data from a live predator‐prey (bacteria and ciliates) system which investigated eco‐evolutionary principles and apply a mathematical model to predict how various transfer volumes and transfer intervals would affect such an experiment. We find not only the ecological dynamics to be modified by both factors but also the evolutionary rates to be affected. Our work predicts that the evolution of the anti‐predator defense in the bacteria, and the evolution of the predation efficiency in the ciliates, both slow down with lower transfer volume, but speed up with longer transfer intervals. Our results provide testable hypotheses for future studies of predator‐prey systems, and we hope this work will help improve our understanding of how ecological and evolutionary processes together shape composition of microbial communities.

## Graphical abstract

Experiments using microbes contribute to our understanding of ecological and evolutionary principles how communities function. Often in experiments those communities are maintained by regular transfers, but details of how that is done often receive little attention. Here, we model experimental data and allow different designs and our findings suggest that this may have substantial impact on the results.

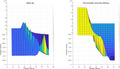

## 1 INTRODUCTION

The composition of microbial communities is sensitive to the environment (Alekseeva et al., 2020; Goldford et al., 2018; Scheuerl et al., 2020), which changes growth of individual species (Bittleston et al., 2020; de Mazancourt et al., 2008) and the interaction with other community members (Fiegna et al., 2015; Fiegna et al., 2015; Gibert & Brassil, 2014). Modifications of the environment can affect predator‐prey systems (Gilpin, 1972), and a stable predator‐prey community may be destabilized due to dwindling densities of a keystone species (Banerjee et al., 2018; Gilljam et al., 2015). For example, a predator may go extinct if the density of the prey becomes too low (Fussmann et al., 2003). Following this, environmental changes can affect community structure and composition and may disrupt vital functions pivotal for ecosystem functioning. Changes of the environment may include the use of antibiotics (Dethlefsen & Relman, 2011) or eutrophication of lake ecosystems (Kearns et al., 2016; Kiersztyn et al., 2019; Kuiper et al., 2015), just as few examples which have been demonstrated to change communities.

A common effect of environmental change is the modification of the mortality rate (Abreu et al., 2019) and for how long the community can grow without further disturbance. These two aspects can be easily implemented in laboratory experiments. In fact, a standard method in experimental studies exploring ecological and evolutionary questions is using microbial communities with periodic transfer to fresh conditions (Hiltunen et al., 2017, 2018; Nair et al., 2019; Scheuerl et al., 2019). In such experiments, two or more species are cultivated in batch culture for a certain period of time, after which a subset of the community is transferred to fresh conditions (Barrick & Lenski, 2013). After initiating each growth cycle using serial‐dilution, the organisms start growing and deplete the available resources. In predator‐prey systems, the prey initially grow fast, but at later stages, when the predators are dense enough, the prey population is consumed. Although this serial dilution does rarely reflect conditions found in nature, these approaches allow estimating population densities and traits undergoing evolution, so various hypotheses can be tested to understand principles. In liquid media that contain all nutrients for rapid cell division, microbes can grow extremely quickly, which makes them suitable study organisms for experiments exploring ecological and evolutionary dynamics over several generations (Buckling et al., 2009). This, however, means that populations reach limiting conditions quickly. To keep the growth conditions constant, populations are commonly either maintained in chemostat systems (Fussmann et al., 2003; Scheuerl & Stelzer, 2019; Stelzer, 2009) or a proportion of the population is transferred to fresh conditions regularly (often between 24 hr and 72 hr) (Fiegna, Scheuerl, et al., 2015; Good et al., 2017; Hiltunen et al., 2017; Lawrence et al., 2012; Scheuerl et al., 2019; Scheuerl & Stelzer, 2017). Diluting a small part of the populations every few days is a classical approach to keep populations constantly growing and to avoid growth plateaus, for example, reaching carrying capacity, once nutrient limitation occurs (Bennett et al., 1990). The two key parameters of dilution, *transfer volume* and *transfer interval*, are often chosen without further investigation. We investigate how dilution, that is transfer volume and transfer interval, affects ecological changes and the speed of grazing resistance/efficiency evolution, by disentangling the two options to realize different dilution terms of a non‐chemostat setting. When batch cultures are regularly transferred to fresh conditions, these are fundamentally different conditions compared to a chemostat system, where medium is replenished on a constant rate, which retains populations at the maximum possible density supported by the settings (Barrick & Lenski, 2013). In batch cultures, populations grow rapidly and exploit the resources, but then experience fresh conditions after transfer to grow rapidly again.

**FIGURE 1:**
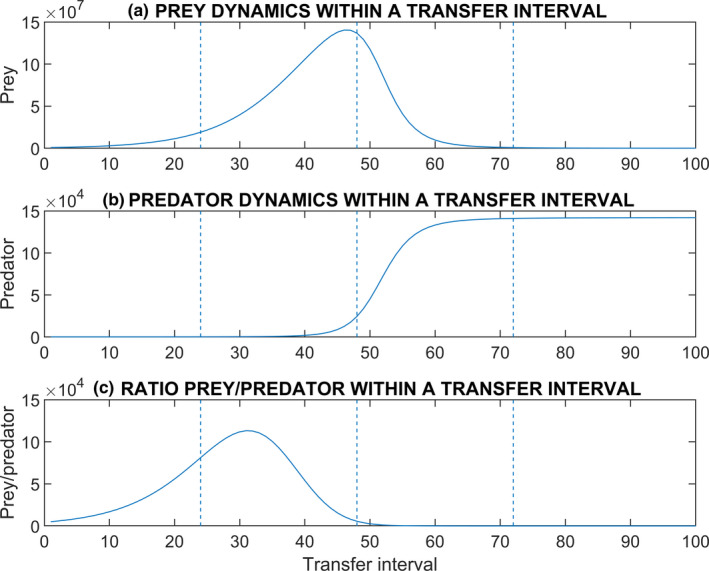
Hypothetical example dynamics of a predator‐prey system within a transfer interval. The abundances of the prey and the predator may differ massively at the time of a transfer depending on the length of the transfer interval. (a) Prey densities; (b) Predator densities; and (c) The ratio of the prey and predator abundances. Three alternative transfer intervals are indicated by vertical lines: 24, 48, and 72 hr

In a community with predator‐prey interaction, theoretically, decreasing the transfer volume to increase dilution (e.g., 1% instead of 10%) results in lower initial densities and prey may initially grow little constrained by predation as predators are rare. Further, prey populations may not be under strong selection to defend because rarely, or only shortly before the next transfer, they encounter predators (Friman et al., 2008; Fussmann et al., 2000; Scheuerl & Stelzer, 2019). Contrarily, extending the transfer interval (e.g., every 48 hr instead of every 24 hr) should increase final densities so that prey and predator encounter each other more often, which may intensify evolutionary changes in the defense of prey. Consider growing bacteria as prey and ciliates as predators for a single growth period (Figure 1). Bacteria will begin growing exponentially until internal density regulation stops this increase. Predation further slows the growth of the prey and may result in a population collapse (Figure 1a). When bacterial densities are high enough, the ciliates will consume the bacterial cells and will increase in density (Figure 1b), this way reducing bacterial densities until ciliates can grow no more due to lack of prey. It can be easily seen that the transfer interval and the transfer volume can both have major impact on the next growth period. If the transfer interval is short, only bacterial densities may be high and ciliate densities may still be neglectable. If the transfer interval is long, ciliates may have already consumed most bacteria, and the next growth cycle is initiated at different densities compared to the previous round. Thus, the transfer interval mainly determines the ratio between prey and predator at each transfer for the next growth period (Figure 1c), whereas transfer volume controls initial conditions for each growth period. Missing in our knowledge is how modification of both factors, transfer volume and transfer interval, together affect ecology and evolution in an experimental predator‐prey community. Experimental tests of ecological and evolutionary dynamics in microbial predator‐prey systems are extremely laborious and applying more than one transfer volume and transfer interval is usually not doable. Theoretical modeling offers a convenient approach out of this dilemma.

Here, we explore experimental data of a predator‐prey experiment from the literature (see reference (Hiltunen et al., 2018)) and apply mathematical modeling to explore multiple modifications of the original protocols. We use a semi‐continuous Lotka‐Volterra model (including dilution of populations at regular intervals) and added equations allowing for co‐evolutionary change of interaction (Kaitala et al., 2020). Expanding our previous model (Kaitala et al., 2020), we report how transfer volume and transfer interval affect predator‐prey communities and expand the prior literature by exploring scenarios impractical in experimental studies. Our theoretical findings suggest that dilution has effects on the community. First, decreasing the transfer volumes, we find that coexistence is threatened, and evolutionary change is limited, while increasing transfer volumes results in more evolution. Second, decreasing transfer interval has similar effects driving populations extinct and decreasing evolutionary rates, while an increase reverses the trend. Our aim was to gain further mechanistic insights into this well‐established predator‐prey system, and thus, we focus in our analysis on the similar scenarios to those of the original study (Hiltunen et al., 2018). In this study, the authors tracked ciliates consuming bacteria, and transferred 1% (transfer volume) of the microorganisms every 48 hr (transfer interval). While the model would allow to simulate a much broader parameter space (e.g., dilution between 0% and 99%), we are missing further information to validate model results. It is worth of noting that the transfer volume or the transfer interval has not been standardized in similar experiments. It is also important to note here that due to the transfer design, it is unlikely to see population cycles as any dynamics may be disrupted during transfers. Finally, we can assume that natural mortality rate is rather low because the transfers in the experiments represent a substantial mortality factor for each of the species. We acknowledge that our model simplifies naturally observed dynamics, but we aim for a model easy to understand even by researcher less familiar with mathematical models but conducting related experiments.

## 2 METHODS

We mathematically modeled the co‐evolutionary predator‐prey interactions of a published study (Hiltunen et al., 2018) applying an ecological Lotka‐Volterra model (Volterra, 1926) modified to explain co‐evolution between the prey and predator (Kaitala et al., 2020; Mougi, 2010; Mougi & Iwasa, 2011). In the experimental study, 1% of the population was transferred after a 48‐hr interval to fresh conditions (Hiltunen et al., 2018). Our model represents the growth period of the experiment, which is initiated newly applying a transfer volume by the end of the transfer interval to obtain a semi‐continuous system.

We use the following modification of the Lotka‐Volterra model

$$\frac{dP\left(t\right)}{\mathrm{dt}}=r_{P}\left(1‐\frac{P\left(t\right)}{K}\right)P\left(t\right)‐aP\left(t\right)Z\left(t\right)$$

$$\frac{dZ\left(t\right)}{\mathrm{dt}}=baP\left(t\right)Z\left(t\right)$$

where the linear growth of the prey is replaced by logistic growth and the natural mortality of the predator is omitted, because of the high dilution in the design. *P* and *Z* denote the prey and predator populations, *r_P_* is the prey growth rate, *K* is the carrying capacity, *a* is the attack rate, and *b* is prey to predator conversion efficiency.

In the co‐evolutionary version, the Lotka‐Volterra model is revised such that the attack rate *a* and the conversion efficiency *b* are functions of auxiliary trait variables *u* and *v* of the prey and predator, respectively (Kaitala et al., 2020; Mougi, 2010; Mougi & Iwasa, 2011). The trait variables have dynamics of their own, the purpose of which is to maximize the fitness of the corresponding species. Thus, the co‐evolutionary model can be presented as follows:

$$\frac{dP\left(t\right)}{\mathrm{dt}}=W_{P}\left(u\left(t\right),v\left(t\right)\right)P\left(t\right),$$

$$\frac{dZ\left(t\right)}{\mathrm{dt}}=W_{Z}\left(u\left(t\right),v\left(t\right)\right)Z\left(t\right),$$

where

$$W_{P}\left(u\left(t\right),v\left(t\right)\right)=r_{P}\left(1‐\frac{P\left(t\right)}{K}\right)‐a\left(u\left(t\right),v\left(t\right)\right)Z\left(t\right),$$

and

$$W_{Z}\left(u\left(t\right),v\left(t\right)\right)=b\left(v\left(t\right)\right)a\left(u\left(t\right),v\left(t\right)\right)P\left(t\right),$$

are the per capita fitness functions of the prey and the predator.

The per capita fitness functions are controlled by the prey and predator trait variables *u*(*t*) and *v*(*t*), respectively. The trait dynamics are assumed to be driven by a selection gradient, which ultimately aims to maximize fitness. The attack rate and the prey to predator conversion efficiency were assumed to be of the form

$$a\left(u\left(t\right),v\left(t\right)\right)=a_{0}\exp\left(c_{1}v\left(t\right)\right)\exp\left(‐gu\left(t\right)\right),$$

$$b\left(v\left(t\right)\right)=b_{0}\exp\left(‐c_{2}v\left(t\right)\right),$$

respectively (Kaitala et al., 2020). Here, $c_{1},c_{2}$, and g are fixed model parameters estimated from the experimental data (see Kaitala et al., 2020).

The evolutionary dynamics of trait variables *u*(*t*) and *v*(*t*), as defined, e.g., by Abrams et al. (1993) and Mougi (2010), are given as follows:

$$\frac{du\left(t\right)}{\mathrm{dt}}=G_{P}\frac{dW_{P}\left(u\left(t\right),v\left(t\right)\right)}{\mathrm{du}}=G_{P}\left[a_{0}g\exp\left(c_{1}v\left(t\right)‐gu\left(t\right)\right)Z\left(t\right)\right],u\left(0\right)=0,$$

$$\frac{dv\left(t\right)}{\mathrm{dt}}=G_{Z}\frac{dW_{Z}\left(u\left(t\right),v\left(t\right)\right)}{\mathrm{dv}},$$

$$=G_{Z}\left[\left(c_{1}‐c_{2}\right)b_{0}\exp\left(‐c_{2}v\left(t\right)\right)a_{0}\exp\left(c_{1}v\left(t\right)‐gu\left(t\right)\right)P\left(t\right)\right],v\left(0\right)=0,$$

where *G_P_* and *G_Z_* are parameters determining the speed of the evolution of the traits. The evolution of the trait variables then determines the evolution of the attack rate $a\left(u,v\right)$ and the prey to predator conversion efficiency $b\left(v\right)$. In the experimental data studied, the ancestral individuals in each species did not have any earlier history of occurring together in a predator‐prey interaction. Thus, the initial values of the traits *u*(0) and *v*(0) are chosen to be equal to 0. Consequently, the initial bacterial and ciliate populations are referred to as “naïve”. Other parameters are estimated from the experimental data presented elsewhere (Hiltunen et al., 2018). The model variables are shown in Table 1 and the parameter values with units are shown in Table 2. For more details about the model, please see our previous study (Kaitala et al., 2020). The produced evolutionary dynamics are potentially more like evolution from standing genetic variation, as traits change continuously. Note also that the bottleneck effect for small transfer volume cannot be investigated using this model because no discrete units are selected.

**TABLE 1:** Model variables and units

*P*	Bacterial density	Bacterial cells/ml
*Z*	Ciliate density	Ciliate cells/ml
*u*	Prey trait	Dimensionless
*v*	Predator trait	Dimensionless

**TABLE 2:** Model parameter values

$r_{P}$	Growth rate of the bacterium	3.3/h
*K*	Carrying capacity of the bacterium	2.58 × 10^8^ Bacterial cells/ml
*a*_0_	Initial value of attack rate	4.2 × 10^–6^ ml/Ciliate cells/h
*b*_0_	Initial value of prey to predator conversion efficiency	5.75 × 10^–4^ Ciliate cells/Bacterial cells
g	Defense value	7.3347
*c*_1_	Offense value	0.8568
*c*_2_	Conversion value	0.4745
*G_P_*	Speed of prey evolution	0.0017
*G_Z_*	Speed of predator evolution	0.0271

We next study effects on ecological and evolutionary dynamics after modifying the transfer volume or transfer interval while maintaining the original estimated model parameters (Kaitala et al., 2020). The initial condition for the prey is 8.56 × 10^7^ Bacterial cells/ml and for the predator 56,800 Ciliate cells/ml. The numerical simulations were performed using ODE solver ode15s in MATLAB R2019a.

### 2.1 Model fit and experimental data

We estimated parameters necessary for our model using data presented in a study exploring ecological and evolutionary dynamics in a live bacteria‐ciliate system (Hiltunen et al., 2018). The experimental data and our model predictions consistently result in coexisting prey and predator populations under these conditions. Prey densities increase over time because anti‐predatory defense evolves and bacteria get less eatable by ciliates (Hiltunen et al., 2018). The predator densities decrease over time as prey becomes better defended against predatory attacks. Coevolution in the predation prevents further decrease in the predator densities (Cairns et al., 2020) with the level of final densities reached after a few transfers and our model is well equipped to capture these dynamics (Kaitala et al., 2020).

## 3 RESULTS

### 3.1 Changing the transfer volume affects ecological and evolutionary dynamics

**FIGURE 2:**
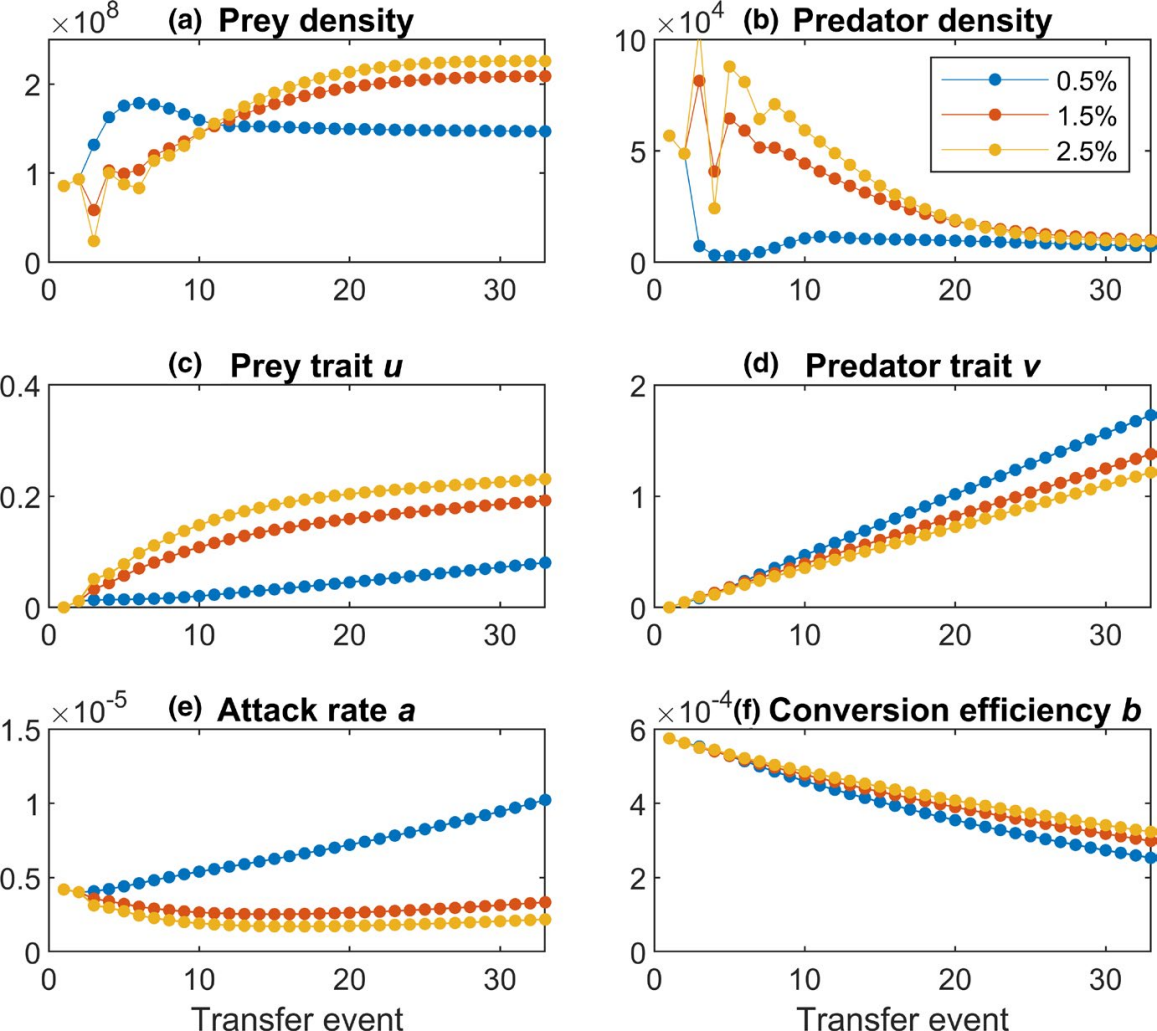
Effect of transfer volume on predator‐prey dynamics. The transfer interval is kept constant at 48 hr. There are 33 transfer events. The transfer volumes are 0.5% (blue), 1.5% (red) and 2.5% (yellow). (a) Bacterial population densities (prey); (b) the ciliate densities (predator); (c) prey trait *u* defining the anti‐predator defense level; (d) predator trait *v*; (e) predator attack rate *a*; and (f) predator conversion efficiency *b*

**FIGURE A1:**
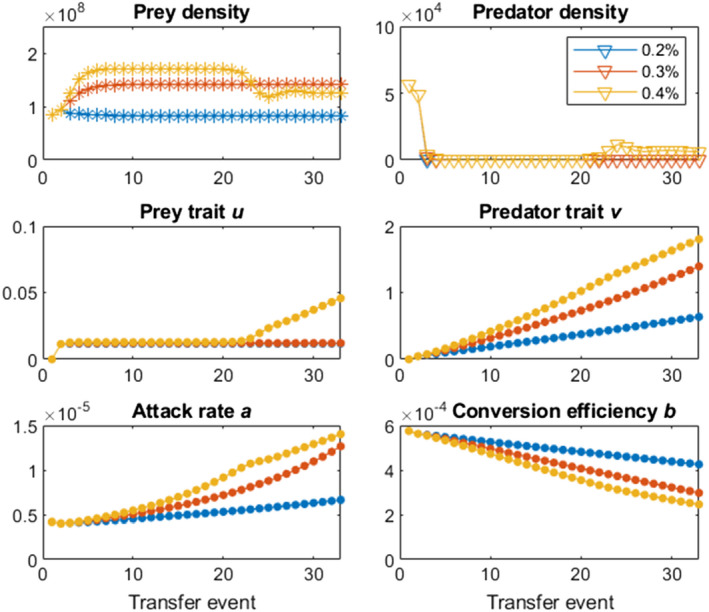
The ecological and evolutionary dynamics under low transfer volumes (0.2%, 0.3% and 0.4%). The experiment is maintained for 33 transfers and at a transfer interval is 48 hr. Blue, red and yellow denote increasing transfer volumes (decreasing dilution)

To explore how dilution by changed transfer volume affects predator‐prey communities, we successively modified the transfer volume in our model (Figure 2) but kept the transfer interval constant at 48 hr. Transferring only 0.5% of the populations (compared to 1.5%) results in reduced bacterial density (Figure 2a) and drives the predator very close to extinction (Figure 2b). Bacterial densities are still observed for a 0.2% transfer volume (Figure A1). Increasing the transfer volume >1.5% has little effect on densities (Figure 2). When dilution is less severe and the next growth cycle is started with higher densities, the initial dynamics seem to fluctuate a bit more in the beginning. However, after a few transfers, the fluctuation in predator‐prey densities fades away and there is no obvious difference between transfer volumes of 1.5% and 2.5% (Figure 2).

Low transfer volumes should release prey from predation pressure because the predator density may be too low to initiate selection high enough to have an effect. Indeed, our results indicate a change in the evolutionary rates. Our model successively predicts that bacterial evolution for increased anti‐predator defense slows down with increasing dilution (Figure 2c). At highest dilution, the anti‐predator prey trait *u* only changes moderately, but when dilution is low (high transfer volume), we see a great change in evolution. On the ciliate side, we see a faster change in predator trait *v* (Figure 2d) and the attack rate *a* (Figure 2e) under low transfer volumes as we would expect when predators are selected for higher attack rate due to reduced encounter events. The conversion efficiency *b* decreases over the course of the experiment, but less under lower transfer volumes (Figure 2f). At the extreme low end of transfer volumes, when only the bacteria survive, anti‐predator defense stops evolving (Figure A1).

**FIGURE A2:**
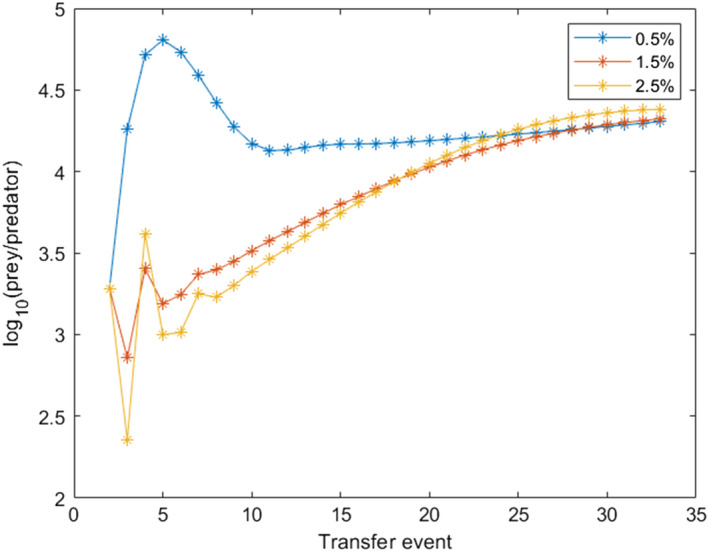
Level of coexistence between bacteria and ciliates in the case studied in Figure 2. The transfer volumes are 0.5% (blue), 1.5% (red) and 2.5% (yellow). The prey to predator ratio differs notably in the beginning of the experiment. The differences level off with time, after 25 transfer events, and with increasing transfer volume

After around 25 transfers, our model predicts that the prey‐predator ratios are the same for all transfer volumes (Figure A2). Before this happens, we see great differences in the bacteria‐ciliate ratios with much more bacteria at highest transfer volume. Predators need a prolonged time to catch up and to establish stable populations. The final ratio, however, seems to be robust against different transfer volumes unless the predator goes extinct.

### 3.2 Changing transfer interval affects ecological and evolutionary dynamics

Because we observed an effect of transfer volume on ecological and evolutionary dynamics in this system, we next addressed the problem whether the transfer interval may have an effect as well. As indicated in Figure 1, unlike transfer volume which keeps ratios sustained, this should affect the bacterial‐ciliate ratio transferred to the next growth cycle. On the ecological side, this means that the transfer interval modifies the initial ratio between bacteria and ciliates for the next growth cycle, which may affect timing when ciliates start to efficiently consume bacteria. On the evolutionary side, anti‐predator defense and attack rate are expected to intensify under longer antagonistic interaction periods.

**FIGURE 3:**
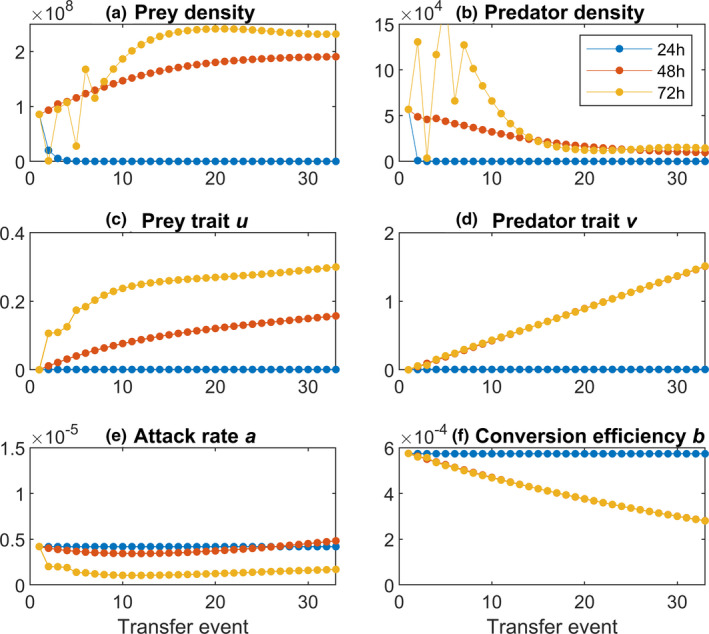
Effect of transfer interval on predator‐prey dynamics. The transfer volume was constant at 1% for all 33 transfers. Transfer intervals are 24 hr (blue), 48 hr (red), and 72 hr (yellow). (a) Bacterial population (prey) and (b) ciliate densities (predator). (c) Prey trait *u*; (d) predator trait *v*; (e) predator attack rate *a;* and (f) predator conversion efficiency *b*

Applying different transfer intervals indeed resulted in various ecological dynamics (Figure 3). The bacterial and ciliate densities are not strongly affected by the length of the intervals (Figure 3a,b). For short transfer interval of 24 hr, both species become extinct. For the intermediate transfer intervals of 48 hr, the bacterial densities steadily increase (Figure 3a), whereas the ciliate densities first steadily decrease, but reach a stable point toward the end of the experiment at low densities (Figure 3b). When the transfer interval is increased to 72 hr, there will be considerable fluctuations in both species in the beginning after which stable coexistence is reached.

When both species become extinct, no evolution will occur (Figure 3c–e). With increasing transfer intervals, we would expect predation activity to intensify, whereas at shorter intervals, predation intensity may be weakened because of low initial densities and reduced encounter rates. A transfer interval less than 48 hr in fact reduces bacterial anti‐predation evolution (Figure 3c), whereas intervals longer than 48 hr result in faster evolution of prey trait *u* in the bacteria (Figure 3c). Predator trait *v* always increases linearly for longer transfer intervals (Figure 3d). The attack rate *a* seems first to decrease slightly, but more under lower dilution (Figure 3e). Again, conversion efficiency *b* linearly decreases, but with no differences between transfer intervals of 48 and 72 hr (Figure 3f).

**FIGURE A3:**
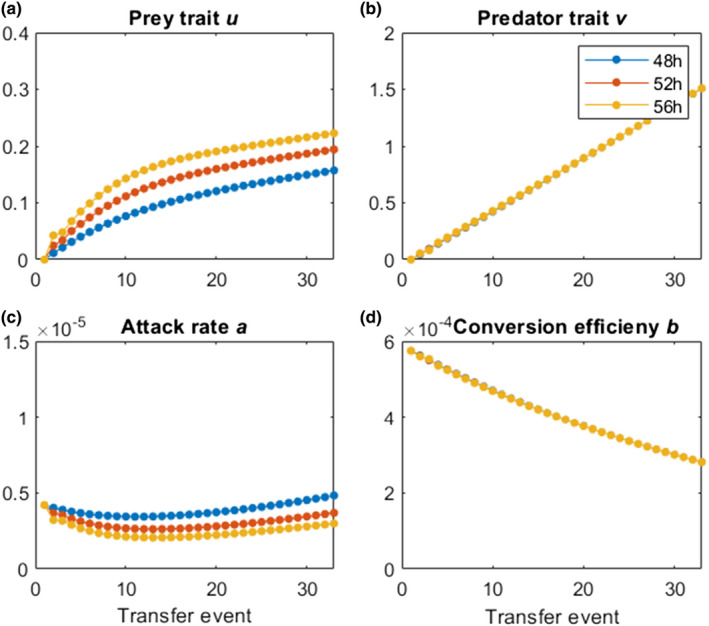
Evolutionary trajectories for slightly increased transfer intervals. (a) prey trait *u*, (b) predator trait *v*, (c) predator attack rate *a* and (d) predator conversion efficiency *b*. Dots in blue, red and yellow, denote increasing transfer intervals of 48, 52 and 56 hr, respectively

We were also interested how evolutionary dynamics are predicted under exceedingly small modifications of transfer intervals. Increasing intervals only slightly (only 2–8 hr) has enhanced impact on evolutionary trajectories (Figure A3). Notably, increasing the interval only initially results in an increase of prey trait *u* in the bacteria, while for predator trait *v*, we see sustained deviations.

### 3.3 Interaction between transfer volume and transfer interval

Because we saw both, transfer volume and transfer interval, to affect ecological and evolutionary dynamics individually, we next investigated how these two parameters interact. For example, a low transfer volume and a long transfer interval both result in increased evolutionary rates and we were interested if the effects are additive and evolutionary rates further increase or are dominant and no further change is observed. To explore this question, we simultaneously modified both factors in our model and tracked the dynamics.

**FIGURE 4:**
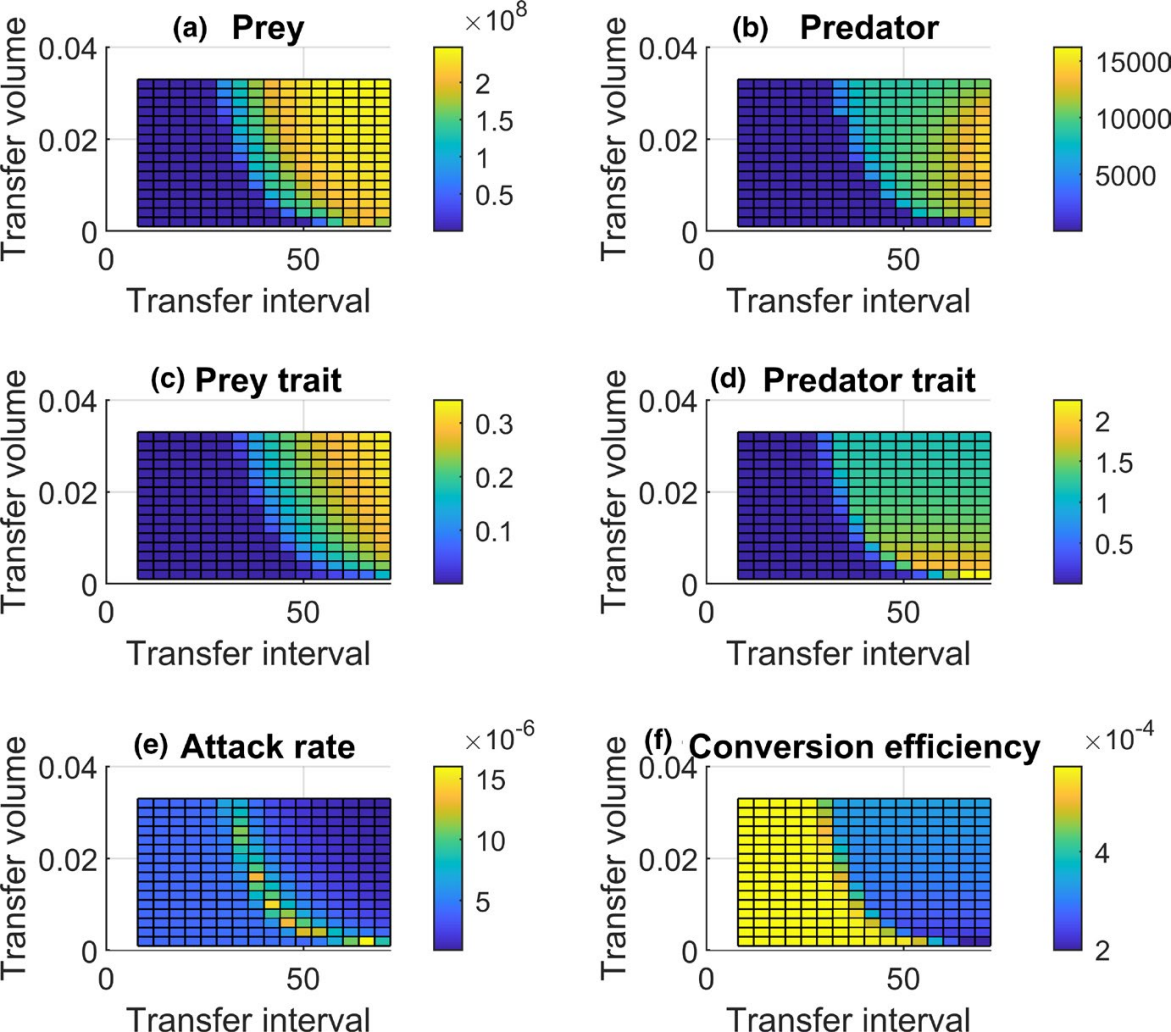
The combined effect of transfer volume and transfer interval on bacterial and ciliate densities and on evolutionary traits. (a) Bacterial densities across various transfer volumes and transfer intervals; (b) Ciliate densities; (c) anti‐predator defense trait *u* of bacteria; (d) predator trait *v*; (e) attack rate *a*; and (f) the conversion efficiency *b* of the ciliates. Vertical bars indicate the value at the end of the experiment (32 transfers)

Our model predicts an interaction between the transfer volume and the transfer interval. Bacterial densities are predicted to be highest at highest transfer volumes and longest transfer intervals (Figure 4a). Contrary to this, we see highest ciliate densities at long transfer intervals, but at intermediate transfer volumes (Figure 4b).

Also, the evolutionary patterns seem to be modified both by transfer volume by and transfer interval (Figure 4c–f). Bacterial anti‐predator defense traits increase continuously and reach highest levels at longest transfer intervals and highest transfer volumes (Figure 4c). For the ciliates, where the maximum species densities are predicted for intermediate transfer volumes and long transfer intervals (Figure 4b), the predator trait *v* initially rapidly increases, but suddenly plateaus off with a peak at low transfer volumes, but long transfer intervals (Figure 4d). The attack rate *a* displays a curved mountain ridge pattern with a moving maximum so that the maximum attack rate is observed when both the transfer volume and the transfer interval increase (Figure 4e). The conversion efficiency *b* is predicted to be stable for a certain transfer volume range, but declines once the transfer interval is too long (Figure 4f).

### 3.4 Sensitivity analysis and human caused impact

Our modeling approach offers additional insights into how sensitive such a predator‐prey experiment is related to protocol changes. In our model, transfer interval and transfer volume are always exact. However, after all, humans are not robots and mistakes can happen. Often, there are slight changes in the protocol maybe because of an occupied autoclave that has not finished in time, researcher forget mixing the microcosms, or pipettors work unprecise which remains unnoticed. To explore how a lack in precision affects the dynamics in such a system, we randomized parameters throughout the simulations.

**FIGURE A4:**
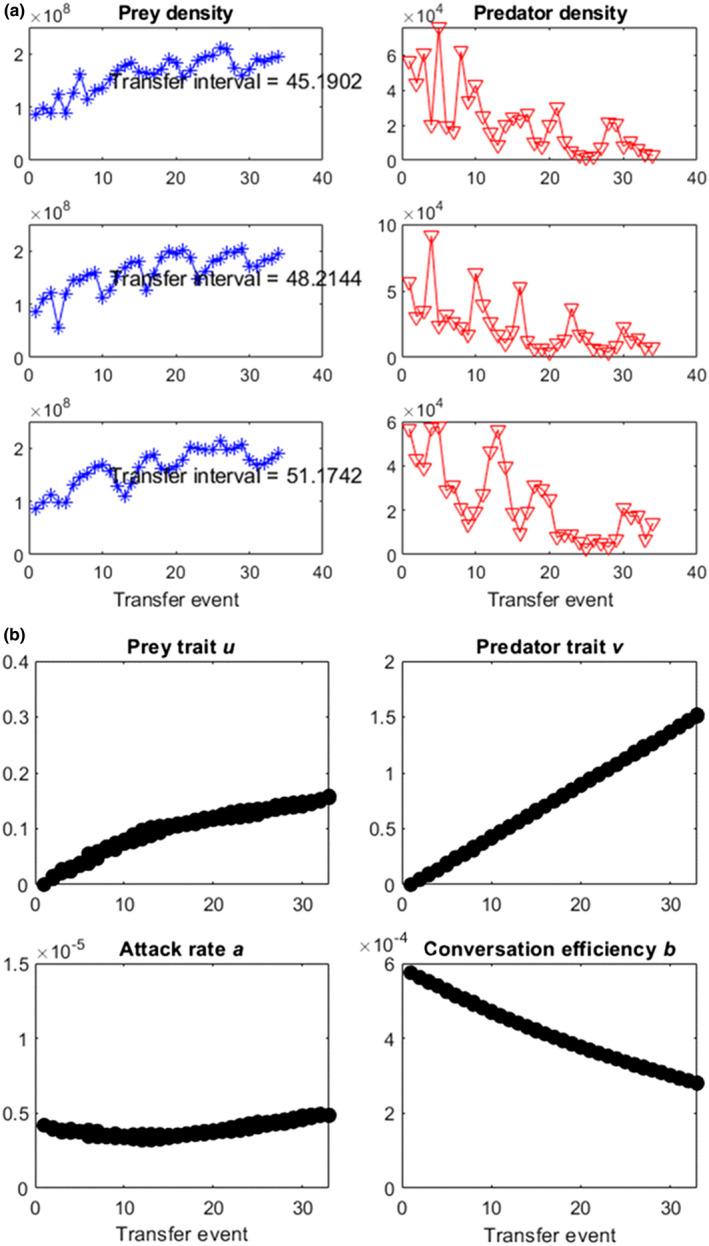
The effect of variation in the transfer intervals. The stochastic sampling intervals are picked from an even distribution defined as 48 hr + (rand − 0.5) × 7 hr, where rand denotes a random even distribution on [0,1]. The expected value of the transfer interval is 48 hr. The sampling intervals are independent. (a) Three replicates of the ecological dynamics for bacteria (blue) and ciliates (red) in the experiments with (b) Evolutionary trajectories of all three replicates presented in (b). The model used a transfer volume of 1%

The first parameter we randomized was transfer interval. For various reasons, every researcher is aware that the transfer interval may deviate from the experimental protocol. So, what would be the effect if the protocol assumes starting a new growth cycle exactly after 48 hr with a transfer activity at 12 p.m., but the transfer happens any time between 9 a.m. and 3 p.m. (Figure A4)? In this scenario, the ecological dynamics begin to display considerable variation (Figure A4a). Particularly the predator densities fluctuate a lot. These dynamics look like predator‐prey dynamics; however, these cycles are not intrinsically induced cycles, but induced by the irregular sampling procedure. The evolutionary trajectories seem to be rather robust for this type of variation (Figure A4b).

**FIGURE A5:**
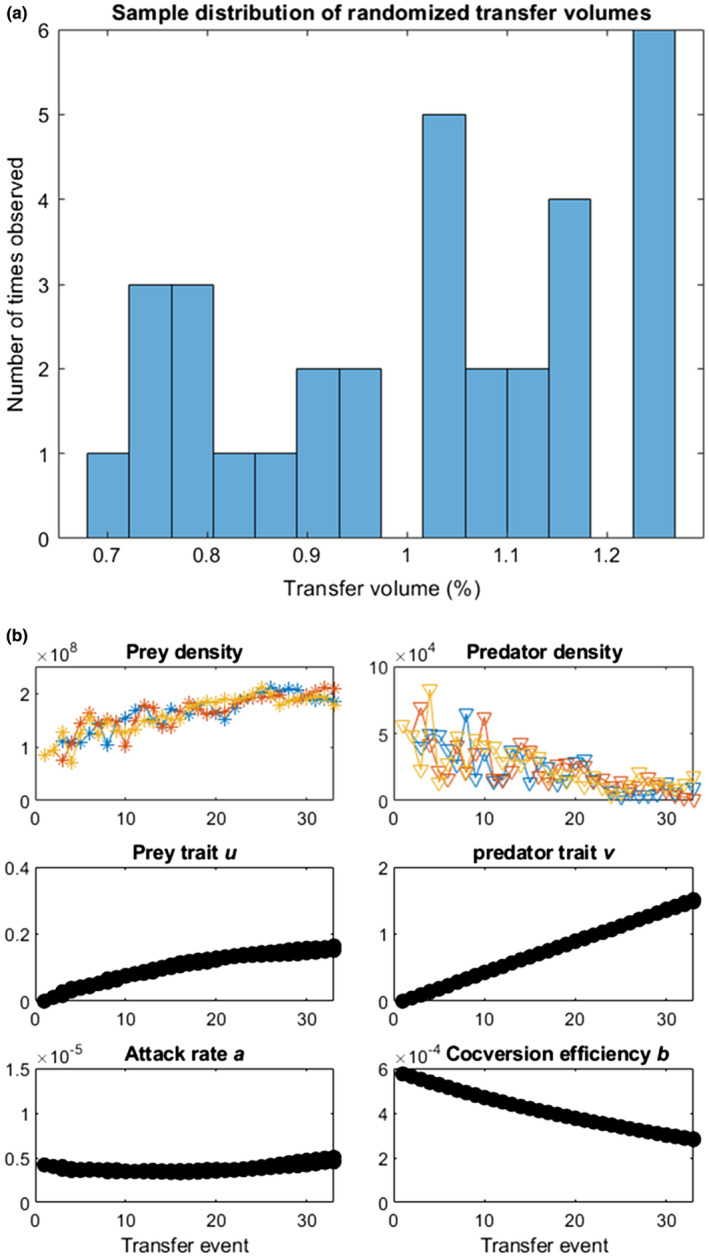
The effect of variable transfer volumes. The transfer volume was picked up from an even distribution between 0.7 and 1.3. (a) An example of the distribution of the randomized transfer volumes in a single experiment. (b) Three examples of the bacterial and ciliate densities in the experiments and corresponding evolutionary dynamics. The evolutionary differences between the three runs were indistinguishable. The model used a transfer interval of 48 hr. Dots in blue, red and yellow, denote increasing bacterial concentrations, respectively

Right before transfer, populations may have patchy distribution when the community is not well mixed, which would result in variation of transfer volumes. We simulated variable transfer volumes by randomizing the transfer volume (Figure A5). The result of this is again that ecological dynamics start fluctuating (Figure A5a). However, evolutionary dynamics are not affected (Figure A5b).

**FIGURE A6:**
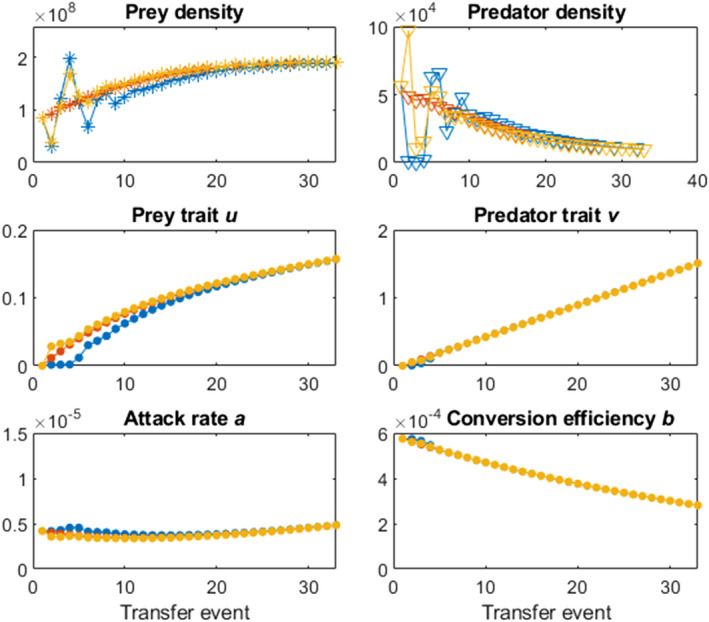
The impact of different initial bacteria concentrations can be seen in the ecological dynamics of the prey and predator, slightly in the prey evolution, but not in the predator evolution. The model used a transfer interval of 48 hr and a transfer volume of 1%. The initial values of the bacterial populations were 56,000, 856,000 and 1,656,000. Dots in blue, red and yellow, denote increasing bacterial concentrations, respectively

**FIGURE A7:**
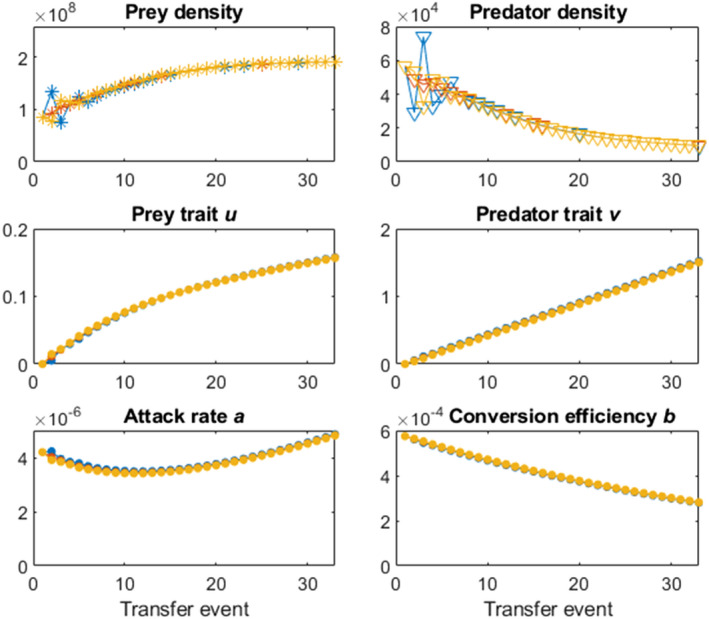
The effect of different initial ciliate concentrations. The model used a transfer interval of 48 hr and a transfer volume of 1%. The initial values of the ciliate populations were 168, 568 and 968. Dots in blue, red and yellow, denote increasing ciliate concentrations, respectively

Another parameter hard to control when starting the experiment is the effect of initial population densities added to the experiment. Researchers commonly estimate the densities of these microorganisms but, of course, the wanted densities can be added roughly only because of the miniature nature of the study system. To simulate this, we started our model assuming different initial densities for bacteria and ciliates. Differences in initial prey densities have little effect on ecological and evolutionary dynamics (Figure A6). Increasing or decreasing the bacterial densities to initiate the experiment is predicted to have no impact. Increasing the initial ciliate density also has little ecological and evolutionary effects (Figure A7). Only the initial predator densities seem to be affected, but after a few growths cycles, this initial effect should be lost.

## 4 DISCUSSION

Experiments using microorganisms offer great insights into evaluating the underlying mechanisms how evolutionary and ecological forces shape communities (Barraclough, 2015; Barrick & Lenski, 2013). However, the specific protocol used in such experiments is likely to have substantial impact on the interpretation of the findings. We used a mathematical model to simulate ecological and evolutionary dynamics of a life predator‐prey system under different transfer volumes and transfer intervals, as this is a common approach in experiments, but the details of the procedures are rarely explored in depth. We feel that our approach making deductions from model predications without further experimental validations turns into a strength, as it allows us to explore many core parameters in fine detail.

Our model predicts that ecological dynamics of experimental bacteria‐ciliate communities including serial transfers are rather robust for changes in transfer volume and transfer interval (Figures 2 and 3). The densities of bacteria and ciliates, however, depend on these parameters under serial transfer design. When transfer volumes become too low or the transfer interval too short, which results in extinction, there are changes in population densities. As could be expected, ciliates become extinct first. A possible explanation is that when the transfer volume is too low, there is no enough prey available and predators are unable to catch enough food to grow rapidly enough to compensate dilution‐induced mortality. While there is potentially enough prey available (we see 1.5 × 10^6^ bacterial cells per ml), these conditions may simply out dilute the ciliates. When there is no enough time to grow, even maximum growth rate may not be high enough to compensate the loss due to dilution. It is further likely that the bacteria and ciliates reach the environmentally imposed growth maximum quickly enough to result in stable densities. Only if dilution results in extinction, this outcome changes, but for most other dilutions, biological drivers, for example, reaching equilibrium, seem to be dominating over experimental procedures.

Our model, however, suggests that evolutionary dynamics are affected by transfer volume and transfer interval together. Increasing the transfer volume is predicted to accelerate anti‐predator defense evolution in the bacteria and attack rate in the ciliates, however, in more complex ways for predators (Figure 2). The transfer interval has also predicted effects, in the sense that longer transfers intensify the evolutionary responses (Figure 3). With decreasing transfer volumes and longer transfer intervals, bacterial defense and ciliate predation both increase, which represents arms‐race dynamics (Brockhurst et al., 2014), as suggested by other studies (Cairns et al., 2020; Kaitala et al., 2020; Klauschies et al., 2016). Our model hereby suggests that there is a pronounced change for evolution from low to intermediate transfer volumes, but less obvious change in evolution from intermediate to high transfer volumes. Why the difference between high dilution and medium dilution seems more pronounced than compared from medium to lowest dilution is unfortunately not straightforward to explain. It could reflect that un‐protected prey benefits a lot when even small defense trait values evolve, whereas at later stages, the effect is not that pronounced anymore, but this is only speculative. A comparison between the transfer volume and the transfer interval suggests that transfer volume may have a little stronger effect on both the ecological and evolutionary dynamics (Figures 2 and 3). However, the ecological dynamics seem to be more sensitive to changes in the transfer interval than for changes in the transfer volume, especially at the beginning of the experiment. Further, we see more variation in the evolutionary trait changes than changing the transfer interval.

Our findings are in agreement with other experiments maintaining bacteria and ciliates at high and low density, which show how nutrient concentration drives evolution of interactions (Friman et al., 2008). An additional advantage of the experimental system we used is that the ciliates and bacteria have not experienced each other before, a situation commonly referred to as “naïve.” Both partners certainly have a long history of predation, but have been maintained in isolation in laboratories for many years and never specifically faced each other. This allows tracking evolutionary changes unbiased to any specific pre‐adaptations. So, we can obtain detailed insights into the starting point how this interaction evolves.

When transfer volume and transfer interval are both simultaneously modified, we see highest predator density at long transfer intervals, but intermediate transfer volumes. This hump‐shaped pattern in the predator density is interesting, albeit hard to explain; thus, we can only speculate again. It could be that under high transfer volumes anti‐predator defense evolution is fastest and thus edible prey may become scarce even when a high bacterial density may be present. This is described by the idea of effective prey biomass, which states that the ratio between edible and inedible prey has effects on population dynamics (van Velzen & Gaedke, 2017, 2018).

Our model predictions are in line with previous findings suggesting effects of increased mortality rates (high transfer volumes) from abiotic change on community structures (Abreu et al., 2019). Increased mortality rates caused by antibiotics affect ecological and evolutionary dynamics in this bacteria‐ciliate system (Hiltunen et al., 2018). Similarly, competition, which also weakens under decreased population sizes of bacteria, interacts with predation and results in changed ecological and evolutionary dynamics (Scheuerl et al., 2019). Our finding that evolutionary trajectories are equally affected compared to ecological dynamics is a bit in contrast with other studies, however. Increased transfer volumes have been shown to result in the modifications of the compositions of bacterial communities (Abreu et al., 2019), thus more on the ecological side. It needs to be mentioned here that Abreu et al. (2019) did not explore evolution, and thus, limited inferences are possible. Our data are also in contrast with a different predator‐prey system, namely rotifers grazing on algae, cultivated in chemostats. In this system, increasing or decreasing the dilution has great impact on the nature of ecological interaction (Fussmann et al., 2000). Changing the dilution shifts the rotifer‐algal densities between equilibrium and stable limit cycle states. However, this system follows a quite different experimental approach, as there is a constant dilution in chemostats. Thus, both protocols, serial batch transfer and chemostats, can hardly be compared. In accordance with our study, the algal population quickly evolves in the form of alternating genotype frequencies of contrasting defense level (Yoshida et al., 2003). Other bacterial studies, inducing high mortality rates at regular intervals, also detect evolutionary changes in interaction (Fiegna, Moreno‐Letelier, et al., 2015; Lawrence et al., 2012), and thus, we think that our findings represent a general pattern.

Whereas evolutionary trajectories look rather clear for the bacteria and are well in line with experimental predictions, the ciliate coevolution is less obvious (Cairns et al., 2020). Observing comparably little evolutionary change across settings in ciliates may be simply because of slower evolution or depend on the fact that the underlying traits are depending on prey dynamics. This may be reflected by the equal ratios seen under different scenarios (Figure A2). Perhaps evolutionary forces are similar across settings when ratios between bacteria and ciliates are little changing. From a biological perspective, this result makes sense, as rate of evolution is expected to decline over time because of imposed costs, which need to be ameliorated before further change can happen.

We, however, also want to mention again that our approach is limited to specific protocols that are based on experiments using regular dilution of batch cultures. Thus, while helpful to explore principles, comparison to natural dynamics is difficult. We call for a careful attention in planning the experimental design when exploring ecological and evolutionary dynamics in microbial communities. Our modeling study suggests that dilution has effects both on ecological patterns and on evolutionary trajectories. Such experiments will detect ecological and evolutionary dynamics, but the magnitudes may depend on the experimental design. We hope that future researchers will take these ideas into account when designing upcoming evolution and ecology experiments.

## CONFLICT OF INTERESTS

The authors declare no conflict of interests.

## AUTHOR CONTRIBUTIONS

**Thomas Scheuerl:** Conceptualization (equal); data curation (equal); formal analysis (equal); funding acquisition (lead); investigation (equal); methodology (equal); validation (equal); visualization (supporting); writing‐original draft (equal); writing‐review & editing (equal). **Veijo Kaitala:** Conceptualization (equal); data curation (equal); formal analysis (equal); investigation (equal); methodology (equal); project administration (equal); software (lead); validation (equal); visualization (lead); writing‐original draft (equal); writing‐review & editing (equal).

## Data Availability

This paper is a theoretical work and does not generate any new experimental or field data. We only use parameter values of the model from ref. (Kaitala et al., 2020). All model results can be found within the figures or the Appendix A. Original experimental data we used to parameterize our model can be found under (https://ars.els‐cdn.com/content/image/1‐s2.0‐S0022519319304643‐mmc1.xlsx).

## References

[ece38065-bib-0001] Abrams, P. A. , Matsuda, H. , & Harada, Y. (1993). Evolutionarily unstable fitness maxima and stable fitness minima of continuous traits. Evolutionary Ecology, 7, 465–487. 10.1007/BF01237642

[ece38065-bib-0002] Abreu, C. I. , Friedman, J. , Andersen Woltz, V. L. , & Gore, J. (2019). Mortality causes universal changes in microbial community composition. Nature Communications, 10, 2120. 10.1038/s41467-019-09925-0 PMC6509412 31073166

[ece38065-bib-0003] Alekseeva, E. , Doebeli, M. , & Ispolatov, I. (2020). Evolutionary adaptation of high‐diversity communities to changing environments. Ecology and Evolution, 10, 11941–11953. 10.1002/ece3.6695 33209261 PMC7663975

[ece38065-bib-0004] Banerjee, S. , Schlaeppi, K. , & van der Heijden, M. G. A. (2018). Keystone taxa as drivers of microbiome structure and functioning. Nature Reviews Microbiology, 16, 567–576. 10.1038/s41579-018-0024-1 29789680

[ece38065-bib-0005] Barraclough, T. G. (2015). How do species interactions affect evolutionary dynamics across whole communities? Annual Review of Ecology, Evolution, and Systematics, 46, 25–48. 10.1146/annurev-ecolsys-112414-054030

[ece38065-bib-0006] Barrick, J. E. , & Lenski, R. E. (2013). Genome dynamics during experimental evolution. Nature Reviews Genetics, 14, 827–839. 10.1038/nrg3564 PMC4239992 24166031

[ece38065-bib-0007] Bennett, A. F. , Dao, K. M. , & Lenski, R. E. (1990). Rapid evolution in response to high‐temperature selection. Nature, 346, 79–81. 10.1038/346079a0 2195353

[ece38065-bib-0008] Bittleston, L. S. , Gralka, M. , Leventhal, G. E. , Mizrahi, I. , & Cordero, O. X. (2020). Context‐dependent dynamics lead to the assembly of functionally distinct microbial communities. Nature Communications, 11, 1440. 10.1038/s41467-020-15169-0 PMC7080782 32188849

[ece38065-bib-0009] Brockhurst, M. A. , Chapman, T. , King, K. C. , Mank, J. E. , Paterson, S. , & Hurst, G. D. D. (2014). Running with the Red Queen: The role of biotic conflicts in evolution. Proceedings of the Royal Society of London B: Biological Sciences, 281, 20141382. 10.1098/rspb.2014.1382 PMC4240979 25355473

[ece38065-bib-0010] Buckling, A. , Craig Maclean, R. , Brockhurst, M. A. , & Colegrave, N. (2009). The Beagle in a bottle. Nature, 457, 824–829. 10.1038/nature07892 19212400

[ece38065-bib-0011] Cairns, J. , Moerman, F. , Fronhofer, E. A. , Altermatt, F. , & Hiltunen, T. Evolution in interacting species alters predator life‐history traits, behaviour and morphology in experimental microbial communities. Proceedings of the Royal Society B: Biological Sciences, 287(1928), 20200652. 10.1098/rspb.2020.0652 PMC7341940 32486984

[ece38065-bib-0012] de Mazancourt, C. , Johnson, E. , & Barraclough, T. G. (2008). Biodiversity inhibits species' evolutionary responses to changing environments. Ecology Letters, 11, 380–388. 10.1111/j.1461-0248.2008.01152.x 18248449

[ece38065-bib-0013] Dethlefsen, L. , & Relman, D. A. (2011). Incomplete recovery and individualized responses of the human distal gut microbiota to repeated antibiotic perturbation. Proceedings of the National Academy of Sciences of the United States of America, 108(Suppl 1), 4554–4561. 10.1073/pnas.1000087107 20847294 PMC3063582

[ece38065-bib-0014] Fiegna, F. , Moreno‐Letelier, A. , Bell, T. , & Barraclough, T. G. (2015). Evolution of species interactions determines microbial community productivity in new environments. ISME Journal, 9, 1235–1245. 10.1038/ismej.2014.215 PMC4409166 25387206

[ece38065-bib-0015] Fiegna, F. , Scheuerl, T. , Moreno‐Letelier, A. , Bell, T. , & Barraclough, T. G. (2015). Saturating effects of species diversity on life‐history evolution in bacteria. Proceedings of the Royal Society B, 282, 20151794. 10.1098/rspb.2015.1794 26378213 PMC4614762

[ece38065-bib-0016] Friman, V.‐P. , Hiltunen, T. , Laakso, J. , & Kaitala, V. (2008). Availability of prey resources drives evolution of predator–prey interaction. Proceedings of the Royal Society B: Biological Sciences, 275, 1625–1633. 10.1098/rspb.2008.0174 PMC2602816 18430643

[ece38065-bib-0017] Fussmann, G. F. , Ellner, S. P. , & Hairston, N. G. Jr (2003). Evolution as a critical component of plankton dynamics. Proceedings of the Royal Society of London. Series B: Biological Sciences, 270, 1015–1022. 10.1098/rspb.2003.2335 12803890 PMC1691339

[ece38065-bib-0018] Fussmann, G. F. , Ellner, S. P. , Shertzer, K. W. , & Hairston, N. G. Jr (2000). Crossing the Hopf bifurcation in a live predator‐prey system. Science, 290, 1358–1360. 10.1126/science.290.5495.1358 11082063

[ece38065-bib-0019] Gibert, J. P. , & Brassil, C. E. (2014). Individual phenotypic variation reduces interaction strengths in a consumer‐resource system. Ecology and Evolution, 4, 3703–3713. 10.1002/ece3.1212 25478159 PMC4224542

[ece38065-bib-0020] Gilljam, D. , Curtsdotter, A. , & Ebenman, B. (2015). Adaptive rewiring aggravates the effects of species loss in ecosystems. Nature Communications, 6, 8412. 10.1038/ncomms9412 26400367

[ece38065-bib-0021] Gilpin, M. E. , & Rosenzweig, M. L. (1972). Enriched predator‐prey systems: Theoretical stability. Science, 177, 902–904. 10.1126/science.177.4052.902 17780992

[ece38065-bib-0022] Goldford, J. E. , Lu, N. , Bajić, D. , Estrela, S. , Tikhonov, M. , Sanchez‐Gorostiaga, A. , Segrè, D. , Mehta, P. , & Sanchez, A. (2018). Emergent simplicity in microbial community assembly. Science, 361, 469–474. 10.1126/science.aat1168 30072533 PMC6405290

[ece38065-bib-0023] Good, B. H. , McDonald, M. J. , Barrick, J. E. , Lenski, R. E. , & Desai, M. M. (2017). The dynamics of molecular evolution over 60,000 generations. Nature, 551, 45–50. 10.1038/nature24287 29045390 PMC5788700

[ece38065-bib-0024] Hiltunen, T. , Cairns, J. , Frickel, J. , Jalasvuori, M. , Laakso, J. , Kaitala, V. , Künzel, S. , Karakoc, E. , & Becks, L. (2018). Dual‐stressor selection alters eco‐evolutionary dynamics in experimental communities. Nature Ecology & Evolution, 12, 1974–1981. 10.1038/s41559-018-0701-5 30455439

[ece38065-bib-0025] Hiltunen, T. , Kaitala, V. , Laakso, J. , & Becks, L. (2017). Evolutionary contribution to coexistence of competitors in microbial food webs. Proceedings of the Royal Society B: Biological Sciences, 284(1864), 20170415. 10.1098/rspb.2017.0415 PMC5647285 29021178

[ece38065-bib-0026] Kaitala, V. , Hiltunen, T. , Becks, L. , & Scheuerl, T. (2020). Co‐evolution as an important component explaining microbial predator‐prey interaction. Journal of Theoretical Biology, 486, 110095. 10.1016/j.jtbi.2019.110095 31783060

[ece38065-bib-0027] Kearns, P. J. , Angell, J. H. , Howard, E. M. , Deegan, L. A. , Stanley, R. H. R. , & Bowen, J. L. (2016). Nutrient enrichment induces dormancy and decreases diversity of active bacteria in salt marsh sediments. Nature Communications, 7, 12881. 10.1038/ncomms12881 PMC5052679 27666199

[ece38065-bib-0028] Kiersztyn, B. , Chróst, R. , Kaliński, T. , Siuda, W. , Bukowska, A. , Kowalczyk, G. , & Grabowska, K. (2019). Structural and functional microbial diversity along a eutrophication gradient of interconnected lakes undergoing anthropopressure. Scientific Reports, 9, 1–14. 10.1038/s41598-019-47577-8 31366993 PMC6668414

[ece38065-bib-0029] Klauschies, T. , Vasseur, D. A. , & Gaedke, U. (2016). Trait adaptation promotes species coexistence in diverse predator and prey communities. Ecology and Evolution, 6, 4141–4159. 10.1002/ece3.2172 27516870 PMC4972238

[ece38065-bib-0030] Kuiper, J. J. , van Altena, C. , de Ruiter, P. C. , van Gerven, L. P. A. , Janse, J. H. , & Mooij, W. M. (2015). Food‐web stability signals critical transitions in temperate shallow lakes. Nature Communications, 6, 7727. 10.1038/ncomms8727 PMC4518252 26173798

[ece38065-bib-0031] Lawrence, D. , Fiegna, F. , Behrends, V. , Bundy, J. G. , Phillimore, A. B. , Bell, T. , & Barraclough, T. G. (2012). Species interactions alter evolutionary responses to a novel environment. PLoS Biology, 10, e1001330. 10.1371/journal.pbio.1001330 22615541 PMC3352820

[ece38065-bib-0032] Mougi, A. (2010). Coevolution in a one predator–two prey system. PLoS One, 5, e13887. 10.1371/journal.pone.0013887 21085473 PMC2976687

[ece38065-bib-0033] Mougi, A. , & Iwasa, Y. (2011). Unique coevolutionary dynamics in a predator‐prey system. Journal of Theoretical Biology, 277, 83–89. 10.1016/j.jtbi.2011.02.015 21354181

[ece38065-bib-0034] Nair, R. R. , Vasse, M. , Wielgoss, S. , Sun, L. , Yu, Y.‐T.‐N. , & Velicer, G. J. (2019). Bacterial predator‐prey coevolution accelerates genome evolution and selects on virulence‐associated prey defences. Nature Communications, 10, 1–10. 10.1038/s41467-019-12140-6 PMC6754418 31541093

[ece38065-bib-0035] Scheuerl, T. , Cairns, J. , Becks, L. , & Hiltunen, T. (2019). Predator coevolution and prey trait variability determine species coexistence. Proceedings of the Royal Society B: Biological Sciences, 286, 20190245. 10.1098/rspb.2019.0245 PMC6532513 31088272

[ece38065-bib-0036] Scheuerl, T. , Hopkins, M. , Nowell, R. W. , Rivett, D. W. , Barraclough, T. G. , & Bell, T. (2020). Bacterial adaptation is constrained in complex communities. Nature Communications, 11, 1–8. 10.1038/s41467-020-14570-z PMC7005322 32029713

[ece38065-bib-0037] Scheuerl, T. , & Kaitala, V. (2020). Mortality and coexistence time both cause changes in predator‐prey co‐evolutionary dynamics. bioRxiv 2020.04.08.031146. 10.1101/2020.04.08.031146

[ece38065-bib-0038] Scheuerl, T. , & Stelzer, C.‐P. (2017). Sex initiates adaptive evolution by recombination between beneficial loci. PLoS One, 12, e0177895. 10.1371/journal.pone.0177895 28575015 PMC5456038

[ece38065-bib-0039] Scheuerl, T. , & Stelzer, C.‐P. (2019). Asexual reproduction changes predator population dynamics in a life predator–prey system. Population Ecology, 34, 210–216. 10.1002/1438-390X.1017 PMC7594307 33149722

[ece38065-bib-0040] Stelzer, C.‐P. (2009). Automated system for sampling, counting, and biological analysis of rotifer populations. Limnology and Oceanography: Methods, 7, 856–864. 10.4319/lom.2009.7.856 PMC2999893 21151824

[ece38065-bib-0041] van Velzen, E. , & Gaedke, U. (2017). Disentangling eco‐evolutionary dynamics of predator‐prey coevolution: The case of antiphase cycles. Scientific Reports, 7, 17125. 10.1038/s41598-017-17019-4 29215005 PMC5719453

[ece38065-bib-0042] van Velzen, E. , & Gaedke, U. (2018). Reversed predator–prey cycles are driven by the amplitude of prey oscillations. Ecology and Evolution, 8, 6317–6329. 10.1002/ece3.4184 29988457 PMC6024131

[ece38065-bib-0043] Volterra, V. (1926). Fluctuations in the abundance of a species considered mathematically. Nature, 118, 558–560. 10.1038/118558a0

[ece38065-bib-0044] Yoshida, T. , Jones, L. E. , Ellner, S. P. , Fussmann, G. F. , & Hairston, N. G. Jr (2003). Rapid evolution drives ecological dynamics in a predator‐prey system. Nature, 424, 303–306. 10.1038/nature01767 12867979

